# Persistence of racial/ethnic and socioeconomic status disparities among non‐institutionalized patients hospitalized with COVID‐19 in Connecticut, July to December 2020

**DOI:** 10.1111/irv.12945

**Published:** 2021-12-06

**Authors:** Geena Chiumento, Kimberly Yousey‐Hindes, Alexandra Edmundson, James L. Hadler

**Affiliations:** ^1^ Connecticut Emerging Infections Program Yale School of Public Health New Haven Connecticut USA; ^2^ Division of Epidemiology, Bureau of Infectious Disease and Laboratory Sciences Massachusetts Department of Public Health Jamaica Plain Massachusetts USA; ^3^ Connecticut Department of Public Health Hartford Connecticut USA

**Keywords:** census, COVID‐19 hospitalization, racial/ethnic disparities, socioeconomic status

## Abstract

**Background:**

COVID‐19 hospitalizations of non‐institutionalized persons during the first COVID‐19 wave in Connecticut disproportionately affected the elderly, communities of color, and individuals of low socioeconomic status (SES). Whether the magnitude of these disparities changed after the initial lockdown and before vaccine rollout is not well documented.

**Methods:**

All first‐time hospitalizations with laboratory‐confirmed COVID‐19 during July to December 2020, including patients' geocoded residential addresses, were obtained from the Connecticut Department of Public Health. Those living in congregate settings, including nursing homes, were excluded. Community‐dwelling patients were assigned census tract‐level poverty and crowding measures from the 2014–2018 American Community Survey by linking their geocoded addresses to census tracts. Age‐adjusted incidence and relative rates were calculated across demographic and SES measures and compared with those from a similar analysis of hospitalized cases during the initial wave.

**Results:**

During July to December 2020, there were 5652 COVID‐19 hospitalizations in community residents in Connecticut. Incidence was highest among those >85 years, non‐Hispanic Blacks and Hispanic/Latinx compared with non‐Hispanic Whites {relative rate (RR) 3.1 (95% confidence interval [CI] 2.83–3.32) and 5.9 (95% CI 5.58–6.28)}, and persons living in high poverty and high crowding census tracts. Although racial/ethnic and SES disparities during the study period were substantial, they were significantly decreased compared with the first wave of COVID‐19.

**Conclusions:**

The finding of persistent, if reduced, large racial/ethnic disparities in COVID‐19 hospitalizations 2–7 months after the initial lockdown was relaxed and before vaccination was widely available is of concern. These disparities cause a challenge to achieving health equity and are relevant for future pandemic planning.

## 1 INTRODUCTION

Coronavirus disease 2019 (COVID‐19), caused from infection with SARS‐CoV‐2, is a highly contagious, viral disease that can lead to severe health outcomes that may require hospitalization and intensive care.^1^ According to COVID‐NET estimates, at the end of 2020, the cumulative incidence rate of COVID‐19 hospitalizations in the United States was 369.3 hospitalizations per 100,000 population.^2^ Hospitalizations are valuable to study from an epidemiological perspective because they are more likely to accurately reflect who is getting infected with COVID‐19 compared with viral testing that can be prone to testing biases.

Over the course of the pandemic, it has become evident that certain people are hospitalized with COVID‐19 at disproportionately higher rates than others, including the elderly and people with underlying health conditions.^3^, ^4^ People of color, particularly Black and African American communities, have also faced an increased risk of COVID‐19 infection and hospitalization compared with non‐Hispanic White communities,^5^, ^6^, ^7^, ^8^, ^9^, ^10^ as have Hispanic and Latinx patients who in some cases have experienced increased in‐hospital mortality.^7^ Additionally, there is increasing evidence that low socioeconomic status (SES) is an important risk factor for hospitalization and thus, antecedent infection.^5^, ^6^ Individual‐level measures of SES are not typically obtained or available through public health surveillance programs, so instead, census tract‐level measures of poverty and crowding from the US Census can be linked to patients' residential addresses as a way to assess SES disparities.^11^ Census‐tract‐based metrics have been valuable to determining the role SES plays in influenza in Connecticut and in other jurisdictions contributing to FluSurv‐Net.^12^, ^13^, ^14^

To date, we are not aware of studies that analyze COVID‐19 hospitalizations and disparities solely among non‐institutionalized individuals in the community (unlike congregate settings which are mostly closed environments) throughout an entire state using public health surveillance data. In Connecticut, the geographical focus of this analysis, disparities in COVID‐19 hospitalizations that occurred during the state's initial “Stay Safe, Stay Home” lockdown period have been previously described but were limited to those in New Haven and Middlesex counties.^10^ In this analysis, we aim to describe Connecticut's statewide trends in COVID‐19 hospitalization among community members after the first, initial wave of COVID‐19 and before the effect vaccinations would have on epidemiology—a time when most individuals had potential for COVID‐19 exposure—in order to help determine the magnitude and persistence of disparities in COVID‐19 hospitalizations. In addition, we compare the magnitude of racial/ethnic and SES disparities from the initial lockdown period^10^ to those found in this analysis.

## 2 METHODS

### 2.1 Surveillance data

We used statewide surveillance data collected by the Connecticut Department of Public Health (DPH) to monitor COVID‐19 hospitalizations beginning on July 1, 2020. Hospitalizations on or after this point were required to be reported to the DPH by hospital staff completing a case report form, which included relevant information such as the patient's age, sex, and race/ethnicity, along with the COVID‐19 case classification, date of admission, whether the patient resided in a congregate setting, and the patient's residential address.

All patients' residential addresses were automatically geocoded by the DPH, assigning each its census tract identification number. For those addresses that could not be automatically geocoded, the DPH manually geocoded them. Addresses unable to be geocoded included those with PO boxes or those deemed erroneous.

### 2.2 Study population

The study population included all Connecticut residents who were hospitalized at an acute care facility with COVID‐19 for the first time between July 1 and December 31, 2020. All hospitalized patients in the final dataset were classified as either confirmed or probable A. Confirmed cases were defined as patients hospitalized within 14 days of a positive polymerase chain reaction (PCR) test for SARS‐CoV‐2. Probable A cases were defined as patients hospitalized within 14 days of a positive SARS‐CoV‐2 antigen‐based test. Probable B cases were excluded (those patients hospitalized with no SARS‐CoV‐2 diagnostic test but with symptoms consistent with the Council of State and Territorial Epidemiologists' COVID‐19 case definition^15^ or an Office of Chief Medical Examiner [OCME] report of a likely COVID‐19 death).

### 2.3 Census data

Area‐based SES measures of poverty and household crowding for each patient were determined by matching each patient's census tract of residence with the corresponding census tract estimate of poverty and crowding from the 2014–2018 American Community Survey (ACS) 5‐Year Estimates from the US Census (https://data.census.gov/cedsci/). Both SES measures were stratified into four levels based on precedent in Connecticut.^9^, ^11^, ^12^ Poverty, defined as the percentage of households living below the federal poverty level, was categorized as very low (<5%), low (5% to <10%), medium (10% to <20%), and high (≥20%). Crowding, defined as the percentage of households with more than one occupant per room, was categorized as very low (<0.9%), low (0.9% to <2.5%), medium (2.5% to <5%), and high (≥5%).

Census tract‐level, total population estimates were obtained from the 2010 Decennial US Census (https://data.census.gov/cedsci/).

### 2.4 Statistical analysis

Although we described the overall epidemiology of COVID‐19 hospitalizations in Connecticut, our analyses placed emphasis on patients who resided in the community, as opposed to congregate settings. Crude and age‐adjusted incidence rates of COVID‐19 hospitalizations were calculated by dividing the case counts by the total population estimates for each age group, gender, race/ethnicity group, poverty level, and crowding level. Age adjustments, used to account for potential age‐related confounding, were based on the 2000 US Standard Population proportions. Chi‐square tests were used to compare hospitalization incidence between demographic and SES strata. Mantel‐Haenszel chi‐square tests for trend were used to determine whether there were significant associations between increasing poverty and crowding levels with hospital incidence, both alone and within age, gender, and race/ethnicity groups.

Additionally, we split these data into two groups: (1) patients residing in New Haven and Middlesex Counties (population: 1,028,153) and (2) patients residing in Fairfield, Litchfield, Hartford, Tolland, Windham, and New London Counties (population: 2,539,394), so that the New Haven and Middlesex County data could be compared with earlier COVID‐NET estimates, which were limited to these two counties. The distribution and age‐adjusted incidence among demographic and SES indicators were calculated and compared between these two county‐based groups to determine if disparities were geographically widespread. Then, for patients residing in New Haven and Middlesex counties, we compared these July through December data with the March through early‐May data previously analyzed by COVID‐NET to determine if trends and disparities in COVID‐19 hospitalizations were persistent throughout the year. Both chi‐square tests and Mantel‐Haenszel chi‐square tests for trend were used for these county‐level analyses. All statistical analyses were performed using SAS version 9.4 (SAS Institute Inc, Cary, NC, USA) and Epi Info version 5.5.3 (Centers for Disease Control and Prevention, Atlanta, GA, USA).

## 3 RESULTS

**FIGURE 1:**
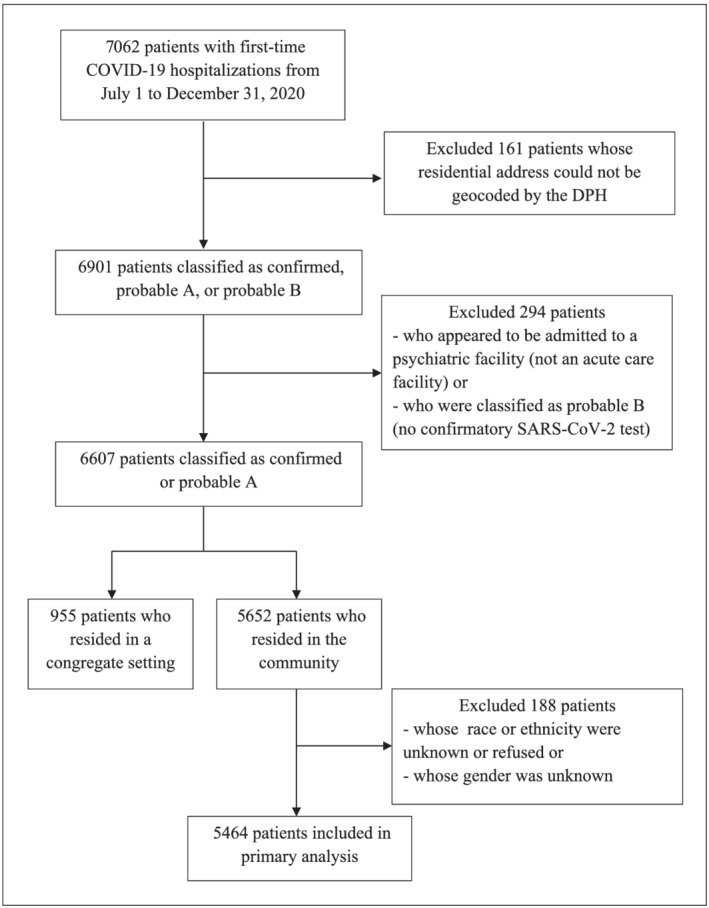
Flow diagram of patients hospitalized with COVID‐19 included or excluded in analyses based on inclusion criteria

There were 7062 first‐time COVID‐19 hospitalizations among Connecticut residents from July 1 to December 31, 2020. Approximately 98% (6901) of patients' residential addresses were successfully geocoded by the DPH. Of these, 294 patients were excluded from analyses because they did not meet this study's criteria and/or were missing data (Figure 1).

### 3.1 Characteristics of all patients hospitalized with COVID‐19

After exclusion criteria, there were 6607 first‐time COVID‐19 hospitalizations between July 1 to December 31, 2020, that were confirmed with a positive molecular or antigen‐based SARS‐CoV‐2 test and had a geocodable residential address (Table 1). Of these, there was a wide range of ages, though only 1.1% (74/6607) of patients were under the age of 18 years. Over half (52.2%) identified their race/ethnicity as non‐Hispanic White, whereas non‐Hispanic Black and Hispanic/Latinx patients represented 12.3% and 21.9% of all, respectively. A total of 5652 (85.5%) cases involved persons who lived in the community, whereas 955 (14.5%) lived in some type of congregate setting (i.e., long‐term care facility, assisted living facility, jail or prison, or group home). The frequency of hospital admissions varied across the 6‐month period, with 78.2% occurring in November and December.

**TABLE 1:** Characteristics of all patients with geocodable residential addresses hospitalized with laboratory‐confirmed COVID‐19 in CT, July to December 2020

Demographic factor	No. of patients	%
Total hospitalized patients	6607	—
Classification		
Confirmed by NAAT	6492	98.3
Probable A	115	1.7
Age (years)		
<18	74	1.1
18–49	1212	18.3
50–64	1649	25.0
65–74	1378	20.9
75–84	1286	19.5
≥85	1008	15.3
Gender		
Female	3237	49.0
Male	3364	50.9
Unknown	6	0.1
Race/Ethnicity		
Non‐Hispanic White	3449	52.2
Non‐Hispanic Black	815	12.3
Non‐Hispanic Asian	84	1.3
Hispanic/Latinx	1450	21.9
Non‐Hispanic Other^a^	606	9.2
Unknown/Refused	203	3.1
Residence		
Community	5652	85.5
Congregate setting	955	14.5
Date of admission		
July 1 to August 31	401	6.1
September 1 to October 31	1038	15.7
November 1 to December 31	5168	78.2

Abbreviation: NAAT, Nucleic Acid Amplification Test.

^a^ Includes Other, Multiracial, American Indian Alaskan Native, and Native Hawaiian and Other Pacific Islander races.

Comparing those living in community with congregate settings, there was a significantly higher percentage of patients aged 75 years or older living in congregate settings (63.4% vs. 29.9%, *P* < 0.001) (data not shown).

### 3.2 Demographic‐based disparities in hospitalization incidence

After excluding 188 (3.3%) patients in the community whose race, ethnicity, and/or gender were unknown, there were 5464 non‐institutionalized patients included in the analysis. Incidence and trends of COVID‐19 hospitalization significantly varied by age and race/ethnicity groups (Table 2). Elderly persons were disproportionately hospitalized; 75‐ to 84‐year‐old and ≥85‐year‐old patients were hospitalized at rates 8.4 (95% confidence interval [CI] 7.70–9.12) and 9.9 (95% CI 9.01–10.95) times higher, respectively, compared with 18‐ to 49‐year‐old patients. There were also significantly higher rates of hospitalization among patients of color, except for non‐Hispanic Asian patients. The age‐adjusted relative rates among non‐Hispanic Black and Hispanic/Latinx cases compared with non‐Hispanic White cases were 3.1 (95% CI 2.83–3.32),and 5.9 (95% CI 5.58–6.28), respectively.

**TABLE 2:** Characteristics, crude and age‐adjusted incidence, and relative rates (RR) for all non‐institutionalized patients hospitalized with COVID‐19 in CT, July to December 2020

Demographic factor	No. of patients (%)	Total pop	Crude incidence/100,000 population	Crude RR	Age‐adjusted incidence/100,000 population	Age‐adjusted RR	95% CI (chi‐square)
Total hospitalized patients	5464	3,567,547	153.2	—	135.9	—	—
Age (years)							
<18	68 (12.4)	816,820	8.3	0.11	8.3	0.11	0.09–0.14
18–49	1125 (20.6)	1,517,378	74.1	Ref	74.1	Ref	—
50–64	1458 (26.7)	727,130	200.5	2.70	200.5	2.70	2.50–2.92
65–74	1153 (21.1)	254,772	452.6	6.10	452.6	6.10	5.62–6.63
75–84	1035 (18.9)	166,602	621.2	8.38	621.2	8.38	7.70–9.12
≥85	625 (11.4)	84,845	736.6	9.94	736.6	9.94	9.01–10.95
Gender							
Female	2657 (48.6)	1,833,851	144.9	Ref	121.8	Ref	—
Male	2807 (51.4)	1,733,696	161.9	1.12	155.0	1.27	1.20–1.35
Race/Ethnicity							
Non‐Hispanic White	2815 (51.5)	2,542,250	110.7	Ref	82.6	Ref	—
Non‐Hispanic Black	743 (13.6)	333,961	222.5	2.01	253.1	3.07	2.83–3.32
Non‐Hispanic Asian	79 (1.4)	133,988	59.0	0.53	92.0	1.11	0.93–1.33
Hispanic/Latinx	1372 (25.1)	478,022	287.0	2.59	488.6	5.92	5.58–6.28
Non‐Hispanic Other^a^	455 (8.3)	—	—	—	—	—	—
Poverty level							
Very low (<5%)	1544 (28.3)	1,420,923	108.7	Ref	90.6	Ref	—
Low (5‐ < 10%)	1302 (23.8)	956,905	136.1	1.25	109.3	1.21	1.11–1.31
Medium (10‐ < 20%)	1325 (24.2)	664,155	199.5	1.84	187.4	2.07	1.91–2.24
High (≥20%)	1293 (23.7)	525,564	246.0	2.26	281.1	3.10	2.88–3.34
Crowding level							
Very low (<0.9%)	2036 (37.3)	1,770,352	115.0	Ref	95.1	Ref	—
Low (0.9% to <2.5%)	1302 (23.8)	839,568	155.1	1.35	132.9	1.40	1.30–1.51
Medium (2.5% to <5%)	1017 (18.6)	496,056	205.0	1.78	195.0	2.05	1.89–2.22
High (≥5%)	1109 (20.3)	461,571	240.3	2.09	269.0	2.83	2.63–3.05

^a^ Includes Other, Multiracial, American Indian Alaskan Native, and Native Hawaiian and Other Pacific Islander race.

### 3.3 SES‐based disparities in hospitalization incidence

**FIGURE 2:**
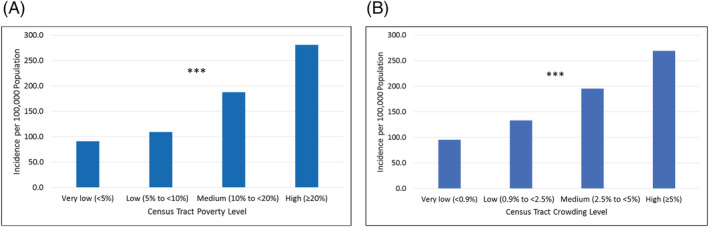
Overall age‐adjusted hospitalization incidence by census tract (A) poverty level and (B) crowding level in CT, July to December 2020. * Chi‐square test for trend *P* < 0.05; ** Chi‐square test for trend *P* < 0.01; *** Chi‐square test for trend *P* < 0.001

When assessing census tract poverty and crowding levels as measures of SES, patients living in high poverty and crowding census tracts were hospitalized at an age‐adjusted rate approximately three times higher (poverty 95% CI 2.88–3.30, crowding 95% CI 2.63–3.05) than patients living in very low poverty and crowding tracts (Table 2). As census tract poverty and crowding levels increased, there were strong and statistically significant trends of increased, age‐adjusted hospitalization incidence (*P* < 0.001 chi‐square for trend for each) (Figure 2A,B).

**FIGURE 3:**
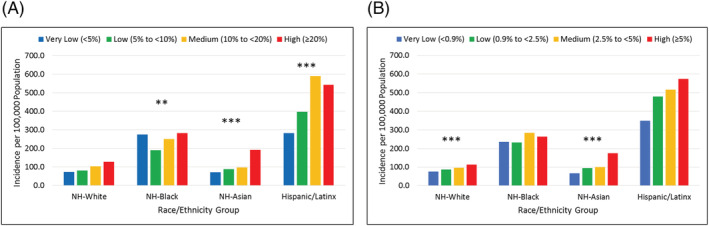
Age‐adjusted hospitalization incidence by census tract (A) poverty level and (B) crowding level and by race/ethnicity group in CT, July to December 2020. NH, non‐Hispanic; * Chi‐square test for trend *P* < 0.05; ** Chi‐square test for trend *P* < 0.01; *** Chi‐square test for trend *P* < 0.001

Across increasing census tract poverty levels, there were statistically highly significant trends (*P* < 0.001) of increasing hospitalization within each race/ethnicity group (Figure 3A), except for non‐Hispanic Blacks (*P* = 0.008).. For increasing census tract crowding levels, statistically insignificant findings were only observed among non‐Hispanic Black patients (*P* = 0.167 chi‐square for trend) (Figure 3B).

### 3.4 County‐level comparisons

Among non‐institutionalized patients, 37.2% resided in New Haven and Middlesex Counties, whereas the remaining 62.8% resided in the other six counties (Table 3A,B). The age‐adjusted incidence in New Haven and Middlesex Counties was approximately 43.6% higher than in the rest of the state; however, percentages of patients and relative incidence of COVID‐19 hospitalization by demographic subgroups were comparable between these two county‐based groups with some exceptions. Disparities were primarily found among patients characterized by low SES after adjusting for age. New Haven and Middlesex County patients living in high poverty and crowding were hospitalized at rates 2.5 (95% CI 2.20–2.81) and 2.1 (95% CI 1.79–2.36) times higher, respectively, than patients living in low poverty and crowding. These disparities were stronger in magnitude for patients of the other six counties, with the high poverty and crowding groups hospitalized at similar rates of 3.4 (95% CI 3.09–3.73) and 3.4 (95% CI 3.13–3.74) times higher than the low poverty and crowding groups, respectively.

**TABLE 3:** Characteristics, crude and age‐adjusted incidence, and relative rates (RR) for non‐institutionalized patients hospitalized with COVID‐19 in (A) New Haven and Middlesex counties and (B) Fairfield, Litchfield, Hartford, Tolland, Windham, and New London counties in CT, July to December 2020

Demographic factor	No. of patients (%)	Total pop	Crude incidence/100,000 population	Crude RR	Age‐adjusted incidence/100,000 population	Age‐adjusted RR	95% CI (chi‐square)
(A) New Haven and Middlesex counties							
Total hospitalized patients	2035	1,028,153	197.9	—	173.2	—	—
Age (years)							
<18	18 (0.9)	228,072	7.9	0.09	7.9	0.09	0.05–0.14
18–49	406 (20.0)	441,329	92	Ref	92	Ref	—
50–64	556 (27.3)	209,159	265.8	2.89	265.8	2.89	2.54–3.28
65–74	451 (22.2)	74,130	608.4	6.61	608.4	6.61	5.78–7.56
75–84	393 (19.3)	49,238	798.2	8.68	798.2	8.68	7.55–9.96
≥85	211 (10.4)	26,225	804.6	8.75	804.6	8.75	7.41–10.32
Gender							
Female	995 (48.9)	532,155	187	Ref	154.2	Ref	—
Male	1040 (51.1)	495,998	209.7	1.12	198	1.28	1.17–1.41
Race/Ethnicity							
Non‐Hispanic White	1148 (56.4)	725,528	158.2	Ref	114.9	Ref	—
Non‐Hispanic Black	350 (17.2)	109,019	321	2.03	368.1	3.20	2.84–3.60
Non‐Hispanic Asian	18 (0.9)	34,140	52.7	0.33	75.3	0.65	0.45–0.98
Hispanic/Latinx	406 (20.0)	137,577	295.1	1.87	543.8	4.73	4.29–5.22
Non‐Hispanic Other^a^	113 (5.6)	—	—	—	—	—	—
Poverty level							
Very low (<5%)	567 (27.9)	366,844	154.6	Ref	121.8	Ref	—
Low (5% to <10%)	513 (25.2)	270,104	189.9	1.23	147.9	1.21	1.06–1.39
Medium (10% to <20%)	427 (21.0)	197,588	216.1	1.4	206.3	1.69	1.48–1.94
High (≥20%)	528 (26.0)	193,617	272.7	1.76	302.6	2.48	2.20–2.81
Crowding level							
Very low (<0.9%)	865 (42.5)	508,471	170.1	Ref	137.7	Ref	—
Low (0.9% to <2.5%)	553 (27.2)	274,286	201.6	1.19	174.9	1.27	1.13–1.43
Medium (2.5 to <5%)	359 (17.6)	144,042	249.2	1.47	240.5	1.75	1.53–1.98
High (≥5%)	258 (12.7)	101,354	254.6	1.5	283.4	2.06	1.79–2.36
(B) Fairfield, Litchfield, Hartford, Tolland, Windham, and New London counties							
Total hospitalized patients	3429	2,539,394	135.0	—	120.6	—	—
Age (years)							
<18	50 (1.5)	588,748	8.5	0.13	8.5	0.13	0.10–0.17
18–49	719 (21.0)	1,076,049	66.8	Ref	66.8	Ref	—
50–64	902 (26.3)	517,971	174.1	2.61	174.1	2.61	2.36–2.87
65–74	702 (20.5)	180,642	388.6	5.82	388.6	5.82	5.24–6.45
75–84	642 (18.7)	117,364	547.0	8.19	547.0	8.19	7.36–9.10
≥85	414 (12.1)	58,620	706.2	10.57	706.2	10.57	9.37–11.92
Gender							
Female	1662 (48.5)	1,301,696	127.7	Ref	108.3	Ref	—
Male	1767 (51.5)	1,237,698	142.8	1.12	137.5	1.27	1.18–1.36
Race/Ethnicity							
Non‐Hispanic White	1667 (48.6)	1,816,722	91.8	Ref	69.2	Ref	—
Non‐Hispanic Black	393 (11.5)	224,942	174.7	1.9	199.2	2.88	2.58–3.21
Non‐Hispanic Asian	61 (1.8)	99,848	61.1	0.67	96.6	1.40	1.13–1.71
Hispanic/Latinx	966 (28.2)	340,445	283.7	3.09	468.7	6.78	6.29–7.29
Non‐Hispanic Other^a^	342 (10.0)	—	—	—	—	—	—
Poverty level							
Very low (<5%)	977 (28.5)	1,054,079	92.7	Ref	79.2	Ref	—
Low (5% to <10%)	789 (23.0)	686,801	114.9	1.24	93.3	1.18	1.06–1.31
Medium (10% to <20%)	898 (26.2)	466,567	192.5	2.08	179.4	2.27	2.06–2.49
High (≥20%)	765 (22.3)	331,947	230.5	2.49	268.7	3.39	3.09–3.73
Crowding level							
Very low (<0.9%)	1171 (34.2)	1,261,881	92.8	Ref	77.5	Ref	—
Low (0.9% to <2.5%)	749 (21.8)	565,282	132.5	1.43	112.6	1.45	1.31–1.60
Medium (2.5% to <5%)	658 (19.2)	352,014	186.9	2.01	176.7	2.28	2.06–2.52
High (≥5%)	851 (24.8)	360,217	236.2	2.55	265.0	3.42	3.13–3.74

^a^ Includes Other, Multiracial, American Indian Alaskan Native, and Native Hawaiian and Other Pacific Islander race.

### 3.5 Time period comparisons

When the 2035 New Haven and Middlesex County patients hospitalized between July 1 to December 31 were compared with 1511 New Haven and Middlesex County patients hospitalized between March 1 and May 8, 2020,^10^ there were significant differences in the magnitude of race/ethnic and SES disparities. The magnitude of the relative age‐adjusted incidence in non‐Hispanic Blacks and Hispanics compared with non‐Hispanic whites decreased from 7.83 and 6.20 during the initial lockdown period to 3.20 and 4.73, respectively, during July through December with no overlap in 95% CIs. Similarly, the magnitude of the disparity comparing the age‐adjusted incidence in the highest to lowest poverty and crowding groups, decreased from 4.67 and 3.35 to 2.48 and 2.06, respectively, with no overlap in 95% CIs (see tab. 1 in Hadler *et al* ^10^ and Table 3B).

## 4 DISCUSSION

Our analysis described the epidemiology of COVID‐19 hospitalizations throughout Connecticut after the initial first wave of COVID‐19 and revealed continued racial/ethnic and SES disparities in hospitalization incidence consequential of community transmission of SARS‐CoV‐2. Despite different incidence in different parts of the state, the magnitudes of the disparities were similar. Although racial/ethnic and SES‐based disparities were high in magnitude across the state, when compared with COVID‐NET data from the “Stay Safe, Stay Home” lockdown period from March to May, they were generally much lower. Additionally, the finding that racial/ethnic disparities in hospitalization were stronger than SES ones during the “Stay Safe, Stay Home” period^10^ remained true throughout the July to December months. Of interest, with influenza hospitalizations in Connecticut, SES disparities have been generally larger than racial/ethnic ones, and both have been lower than the ones found in this analysis.^12^, ^14^

We postulate several explanations for the disparities in COVID‐19 hospitalizations found in this analysis. From March to May 2020, adult, public‐facing essential workers (e.g., health aides, childcare workers, bus drivers, cashiers, factory workers, farm workers, and custodial staff),^16^ disproportionately Black and Hispanic, many without personal protective equipment (PPE), were exposed occupationally, bringing infection into their home and largely segregated neighborhoods, resulting in the high racial/ethnic disparities seen not just in working age adults but also across all age groups. From July to December, with a lifting of restrictions on non‐essential businesses, gatherings, and activities outside the home including camps, sports, and school, a broader spectrum of the population left their homes than during the initial lockdown, resulting in more diversity of potential exposure across age, racial/ethnic, and SES groups. In addition, PPE shortages were largely resolved. These may account in part for the smaller disparities seen July through December than found during the initial wave, a trend that was also observed nationally.^17^

However, despite being smaller, substantial racial/ethnic disparities persisted and remained larger than those seen in Connecticut for influenza hospitalizations. These disparities were particularly large for Hispanic and Latinx patients and among them, had a strong association with household crowding, suggesting large households with transmission within them. Although the dynamics of higher levels of transmission in communities of color during this more open time are otherwise not entirely clear, there was likely a continuing occupational component to it: jobs that could not be done from home with workplace exposure and subsequent household and local community transmission. In addition, essential workers often face economic vulnerability not only due to low wages but also due to these jobs sometimes being part‐time. Individuals who are not able to work from home may also need to use daycare for their children, as daycares have been shown to be a facilitator of SARS‐CoV‐2 transmission from children to their families.^18^ With household crowding as an additional obstacle for isolation and quarantine practices, one new infection can be quickly amplified and reach those who are medically more vulnerable.

The historic cause underlying the occupational, household, and community transmission dynamics is the systemic racism that has denied opportunity, generational education and wealth, and community integration to people of color, particularly non‐Hispanic Black and Hispanic/Latinx patients. In addition, discrimination causes social and economic stress that is associated with a higher frequency and severity of chronic health conditions such as obesity, diabetes and heart disease that predispose to more serious COVID‐19. Furthermore, people of color, especially those of low SES or living in low‐income neighborhoods, may have inadequate access to care, which might result in delayed medical attention and increase an individual's chances of being hospitalized.^9^ For immigrants and undocumented individuals, fear of culturally incompetent providers, language barriers, or deportation may also result in apprehension towards seeking care until their condition becomes critical.^9^

Our findings have important implications for pandemic planning in Connecticut and, likely, other states. It is necessary to understand why marginalized communities (people of color, those living in poverty or crowding) have been initially disproportionately affected by COVID‐19 morbidity so that the proper steps can be taken ahead of time to minimize the impact of another respiratory virus with pandemic potential. The racial and ethnic disparities in COVID‐19 hospitalizations are also likely to persist and even be exacerbated in the vaccine era given the differential COVID‐19 vaccination rates among people of color compared with White people seen in an overwhelming majority of states.^19^

### 4.1 Strengths and limitations

This analysis had several noteworthy strengths and limitations. Its strengths were that it used data from all reported hospitalizations in Connecticut including those from active surveillance in two COVID‐NET counties, New Haven and Middlesex,^20^ examined the epidemiology of community transmission not obfuscated by institutional transmission, and used data from hospitalizations rather than positive tests. However, because this study relied on public health surveillance data, there were missing data components from the initial case reporting, resulting in several “unknowns” for race/ethnicity, type of residence, and ICU admission, in which further analysis could not be done. In addition, surveillance was not active in six counties, and there may have been some underreporting of hospitalizations, potentially contributing to the differences in incidence between the two regions in this analysis. The census tract‐level poverty and crowding measures only characterize SES at the neighborhood level and do not necessarily apply to all individuals or households, although neighborhoods are considered a social determinant of health. Further, the ACS, from where the poverty and crowding measures were obtained, is also based on random sampling of the population, with the potential for misclassification of poverty and crowding levels in some census tracts. Grouping them into four categories, however, likely minimized the potential for bias in misclassification. Most importantly, we were unable to exclude those living in institutions from census tract denominators, leading to community rates that were underestimates, particularly for the age groups living in institutional settings (long‐term care facilities, corrections, etc.).

## 5 CONCLUSION

COVID‐19 hospitalizations have affected various populations throughout Connecticut. Even after Connecticut began to open after the first wave, the elderly, people of color, and those living in census tracts characterized by high poverty and crowding levels remained disproportionately hospitalized compared with younger adults, non‐Hispanic Whites, and individuals of higher SES. Factors related to the consequences of long‐standing systemic racism likely account for persistent disparities in COVID hospitalizations by race and ethnicity. These need to be considered when planning for the response to a future pandemic caused by a communicable virus.

## AUTHOR CONTRIBUTIONS

**Geena Chiumento:** Conceptualization; data curation; formal analysis; investigation; methodology; validation; visualization. **Kimberly Yousey‐Hindes:** Conceptualization; data curation; formal analysis; investigation; methodology; project administration; supervision; validation; visualization. **Alexandra Edmundson:** Conceptualization; data curation; formal analysis; investigation; methodology; validation; visualization. **James Hadler:** Conceptualization; formal analysis; investigation; methodology; resources; supervision; validation; visualization.

## CONFLICT OF INTEREST

The authors have no conflict of interest.

### PEER REVIEW

The peer review history for this article is available at https://publons.com/publon/10.1111/irv.12945.

## Data Availability

The surveillance data that were used in this analysis and that support the findings are the property of the State of Connecticut Department of Public Health (CDPH). These data could be made available from the CDPH with a satisfactory Human Investigations Committee proposal.
